# Ramifications of Atmospheric Humidity on Monsoon Depressions over the Indian Subcontinent

**DOI:** 10.1038/s41598-018-28365-2

**Published:** 2018-07-02

**Authors:** Himadri Baisya, Sandeep Pattnaik, Vivekananda Hazra, Anshul Sisodiya, Deepika Rai

**Affiliations:** School of Earth, Ocean, and Climate Sciences, Indian Institute of Technology Bhubaneswar, Odisha, India

## Abstract

In this study, a comprehensive investigation is carried out to examine the sensitivity of tropospheric relative humidity (RH) on monsoon depressions (MDs) under a changing climate regime through surrogate climate change approach over the Indian region. Composite analysis of four MDs show a persistent warming (RH2+) and cooling (RH2−) throughout the troposphere in the sensitivity experiments. In-depth analysis of a MD over the Arabian Sea (AS) exhibits sustained warming for RH2+, which is accredited to 2.6% increase in stratiform clouds accounting for 13% increment in heating, whereas 5% increment in convective clouds hardly contribute to total heating. Frozen hydrometeors (graupel and snow) are speculated to be the major contributors to this heating. Stratiform clouds showed greater sensitivity to RH perturbations in the lower troposphere (1000–750 hPa), albeit very less sensitivity for convective clouds, both in the lower and mid-troposphere (700–500 hPa). Precipitation is enhanced in a moist situation (RH2+) owing to positive feedbacks induced by moisture influx and precipitation efficiency, while negative feedbacks suppressed precipitation in a dry troposphere (RH2−). In a nutshell, it is inferred that under moist (dry) situations, it is highly likely that intense (weak) MDs will occur in the near future over the Indian region.

## Introduction

In an ever-changing climate with a consistently increasing trend in the global mean temperature, it is apparent that the water holding capacity of the atmosphere will increase at a rate governed by the Clausius-Clapeyron (CC, ~7% °C^−1^) relationship^1,2^. Global land and ocean temperatures in 2016 set a record by overshooting the 1981–2010 average by 0.45° and 0.56 °C respectively, and as a consequence specific humidity (SH) peaked, reaching a record high well above the long-term average^3^. Further, RH is projected to remain nearly constant with an increase in SH. The differential heating of land and ocean has been attributed for a small decrease in the near-surface RH over most land areas with exceptions over parts of Africa and the Indian subcontinent^4^. Dai^5^ documented similar trends from *in situ* observations (1975–2005) with exceptions over the central and eastern United States, India, and western China with RH increase ranging from 0.5–2% decade^−1^. It is inferred that this change is a result of increased RH coupled with moderate warming and enhanced low-level clouds during the analysis period.

The earth’s radiation budget is significantly affected by the presence of water vapor, owing to the absorption of radiation that contributes to changes in the water vapor feedback^6–8^. A 10% increase in RH in the upper troposphere led to ~1.4 Wm^−2^ of radiative forcings^9^. It is found that, if RH distribution is specified instead of absolute humidity, water vapor feedback to climate sensitivity doubled and the atmosphere took twice the time to reach radiative convective equilibrium^10^. Further, studies demonstrated that in Deep Convective Systems (DCS), the convective cores bear the heavy precipitation with widespread rain in the stratiform region; the non-precipitating anvil canopy is dominant in the atmospheric radiation budget due to their sheer spatial coverage^11^. DCS that last more than 6 hours have 50% more mid-tropospheric RH compared to short-lived systems, whereas, a dry mid-tropospheric profile can lead to suppressed deep convection in favor of a shallow convective regime^12,13^. It was also found that an improved RH at the initial time in the model can bring better skills of MDs rainfall predictability (up to day 2) over the Indian region^14^.

Over the Indian subcontinent, the summer monsoon accounts for ~80% of annual precipitation which is crucial for an agrarian society like India^15^. On an average, out of ~14 low-pressure systems that develop during the monsoon season, about 50% develop into depressions^16^. Some concerns have been cited in recent literature regarding a decreasing trend in the number of monsoon depressions due to a decline in the mid-tropospheric RH and moisture flux convergence, weakening the low-level jet^17–20^. Recent studies have also cautioned the use of reanalysis data for trend analysis of monsoon depressions^19,21^. A recent study found a threefold increase in extreme rainfall events over central India during 1950–2015. They attributed it to an increased variability in the low-level monsoon westerlies over the Arabian Sea (AS) driving surges of moisture supply, leading to extreme rainfall episodes across the entire central subcontinent^22^. Past studies have demonstrated that there is an increase in the moisture content of the atmosphere over the Indian region^23^, and this rise is attributed for the increasing trend in extreme rainfall events over central India^24,25^. In addition, studies have showed that an increase in surface warming leads to a rise in the moisture content of the atmosphere over the Indian region^26,27^. Hunt^28^ confirmed from a composite of 106 depressions over the BoB that there is a high resemblance between RH and cloud cover in the south western quadrant of MDs with a precipitation maxima.

Even though the Global Climate Models (GCMs) project an increase in the intensity and frequency of heavy precipitation events, the crude resolution and inability to resolve the sub-grid scale processes pose huge uncertainties in the projected trends^29^. An inadequate representation of monsoon low-pressure systems in GCMs have been held accountable for a dry bias in the central Indian region in the Coupled Model Intercomparison Project-5^30,31^ (CMIP5). Trend analysis of RH using European Centre for Medium Range Weather Forecast (ERAI)^32^ data for the last 39 years (1979–2017) showed that the mid-tropospheric RH (700–500 hPa) has increased about 2% over the Indian subcontinent. It is also noted that the spatial trend of RH for ERAI is well correlated to the Atmospheric Infrared Sounder (AIRS) observation data for a period 2003–2017 (Supplementary Fig. S2a). Therefore, it is extremely pertinent to examine the response of moisture content to the synoptic scale intense rain bearing monsoon low-pressure systems (i.e. MDs) in a changing climate scenario over the Indian region.

**Figure 1:**
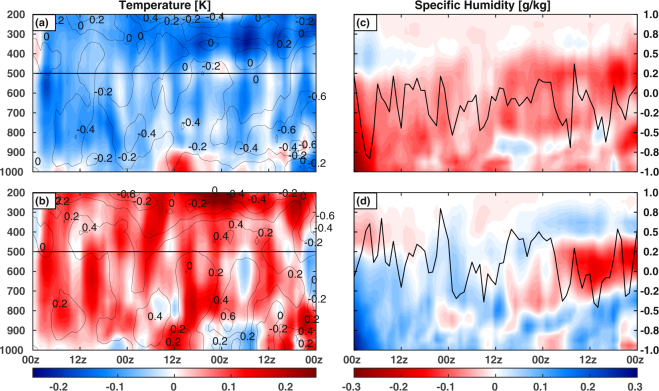
Composite Temperature (K) difference (EXP – CTL) overlaid with wind magnitude (m/s) difference contours for (**a**) RH2−, (**b**) RH2+, and SH (g/kg) difference overlaid with precipitation (mm/h) difference for (**c**) RH2−, and (**d**) RH2+. The solid line at 500 hPa shows the dividing zone between perturbation and compensation of RH in the initial condition. Figures are prepared with MATLAB 2015b (www.mathworks.com).

Surrogate climate change approach acts as a viable tool to investigate the response of a parameter like RH under a changed climate regime to the system of interest, understanding physical processes and parameterization tuning^33–35^. Here, the surrogate climate change approach is adopted to probe the impacts of a changing climate on MDs characteristics arising from the Bay of Bengal (BoB) and the AS with a realistic perturbed RH in the initial state of the model that can be expected in the near future. All the results discussed in the following sections are for the inner domain, unless specifically defined. Figures 1 and 3 represent composite plots [Supplementary Text] of 4 MDs, and rest of the figures is for case 4.

## Results and Discussions

### Temperature and Humidity

The immediate effect of perturbed RH is seen in the temperature field. Following the CC relationship, a 2% increase (decrease) in RH results in ~0.25 °C increase (decrease) in the temperature field. As seen in the difference plots between the experiments (EXP) and the reference run (CTL, Fig. 1a,b), a sharp temperature contrast exists near the 500 hPa region during the initial 3 hours of simulation. Since RH2+ has a positive RH perturbation from the surface to 500 hPa with a compensating effect on the layers above, a rise in temperature is seen only till this level with a reduction in the layers above it, and vice-versa for RH2−. A unique thing to be noted is that after 3 hours of simulation, RH2+ shows a robust increase in temperature throughout its domain, whereas cooling is noted for RH2− predominantly above 500 hPa. This is indicative of other sources of heat (sink) that might be responsible for a sustained enhanced (diminished) temperature profile throughout the simulation. The wind field difference (Fig. 1a,b) reveal a weakening of intensity in RH2− (−0.2 ms^−1^), and strengthening in case of RH2+ (+0.2 ms^−1^) which could be a consequence of the weaker (stronger) temperature gradients in RH2− (RH2+).

Figure 1(c,d) show the composite differences in SH between each experiment and CTL, overlaid with precipitation differences. In general, it found that for RH2− there is a strong dry bias throughout the atmospheric column (−0.3 g/kg) and vice versa for RH2+. However, there is a reduction in the moisture holding capacity of the troposphere in RH2+ after 36 hours of simulation. The gradual decrease of moisture in RH2+ could be due to the fact that all the simulated depressions moved inland by day 2, thus depriving them of moisture from the open seas. Enhanced precipitation scenarios are also seen throughout the simulation period for RH2+ as opposed to subdued conditions for RH2−.

Comprehensive examination of case 4 (Supplementary Fig. S3) showed that until the depression was over the AS, unavailability of moisture both locally and through advection kept SH levels low in case of RH2−. A reduction in initial RH in case of RH2− leads to enhanced evaporation over the sea for the first 12 hours of simulation (Supplementary Fig. S5), thus reducing the RH difference in the troposphere. Further, as the system made landfall (1200 UTC 23 June), a drier troposphere over land facilitated moisture influx (149.18 mm/day). On the other hand, RH2+ starts losing moisture after 36 hours. The added moisture tends to suppress evaporation in the first 12 hours over the AS. The weaker moisture gradient between the sea surface and the near-surface atmosphere restrains evaporation. On day 2, as the depression intensified, evaporation rates increased for RH2+ over the AS, and this can be attributed to stronger winds over the AS compared to RH2− (Supplementary Figs S6, S11). On day 3 when the depression was completely inland, evaporation over land spiked due to the influx of moisture coupled with stronger winds along the storm track in RH2− (2.19 mm/day), whereas it dropped for RH2+ (2.14 mm/day). The rainfall from CTL and experiments are validated against Global Precipitation Mission^36^ (GPM) half hourly dataset, and it is found that the model captures the propagation reasonably well, however, the model simulates the precipitation peaks earlier than GPM (Supplementary Fig. S9).

### Vertical Eddy Flux (VEF)ss

In order to ascertain the persistent cooling (heating) in RH2− (RH2+), VEF is calculated as proposed by Yanai and Johnson^37^ for case 4 only, suspecting clouds to be a major contributor in transporting heat to the upper levels in the troposphere. VEF is defined as

1 $${Q}_{1}-{Q}_{2}-{Q}_{R}=-\,\frac{\partial }{\partial p}\overline{{h}^{\text{'}}{\omega }^{\text{'}}},$$

where *Q*_1_ and *Q*_2_ are the apparent heat source and moisture sink respectively, and *Q*_*R*_ is the heating due to radiation. The term on the right-hand side of equation (1) represents the vertical eddy transport of total heat or VEF and acts as a proxy to measure cumulus convection^38,39^. The perturbations are calculated by taking mean over 30 minutes time period and units are converted to kelvins per day^40^. $\omega$ is vertical *p* velocity, and *h* is moist static energy given as

2 $$h=\,{C}_{p}T+gz+{L}_{v}q$$

where *C*_*p*_ is the specific heat capacity at constant pressure (J/kg/K), *T* is temperature (K), *L*_*v*_ is latent heat of vaporization (J/kg/K), *q* is water vapor mixing ratio (kg/kg), *g* is gravitational acceleration (ms^−2^), and *z* is elevation (m). Collocating data obtained from VEF and cloud categories from rain type algorithm^41^ (RT) enabled us to quantify the amount of heat released by each cloud type. In this study, VEF associated with convective and stratiform clouds only are addressed, owing to their spatial coverage and associated heating signatures.

**Figure 2:**
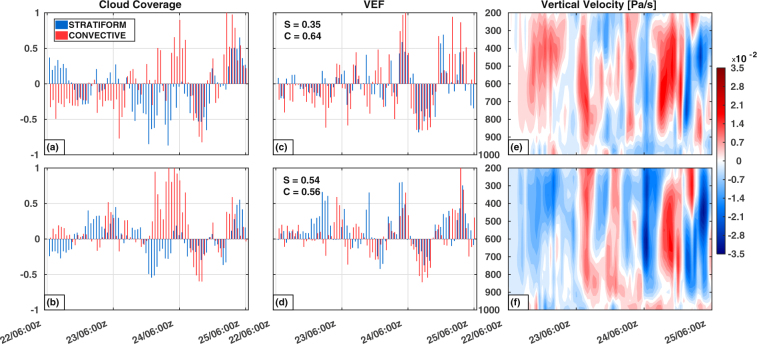
Normalized percentage change in cloud coverage with respect to CTL for (**a**) RH2−, and (**b**) RH2+. (**c**,**d**) As in (**a**,**b**), but for VEF. Each value in the series is normalized by the absolute maxima of stratiform or convective values in that series. S and C represent the correlation coefficients between cloud coverage and VEF for stratiform and convective clouds respectively. Domain averaged vertical *p* velocity (Pa/s) difference for (**e**) RH2−, and (**f**) RH2+. Negative values of vertical velocity signify stronger updraft in the experiment. Figures are prepared with MATLAB 2015b (www.mathworks.com).

**Figure 3:**
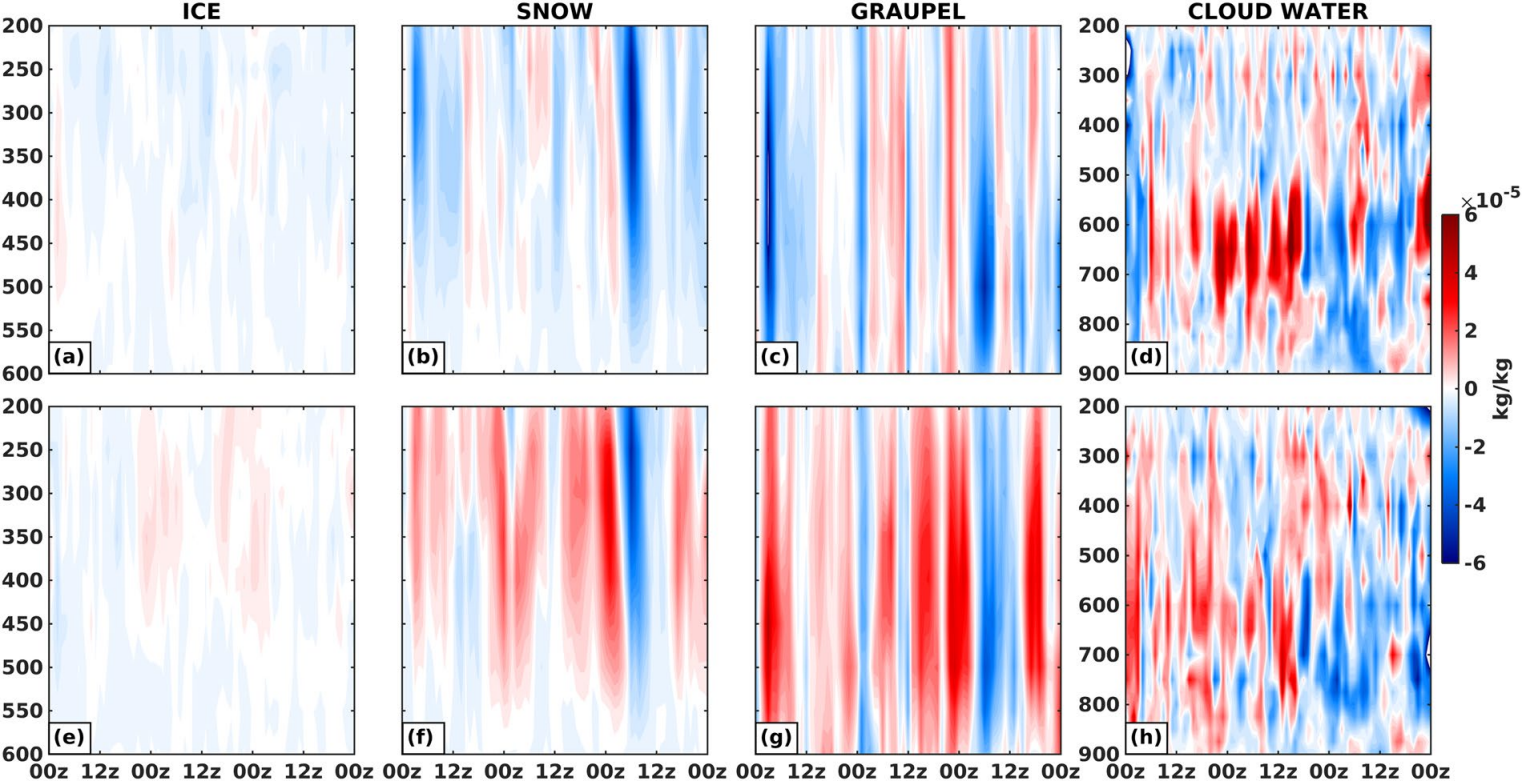
Domain averaged composite plots for hydrometeor mixing ratio (kg/kg) difference (EXP – CTL) in RH2− for (**a**) ice, (**b**) snow, (**c**) graupel, and (**d**) cloud water. (**e**–**h**) same as (**a**–**d**) but for RH2+. Figures are prepared with MATLAB 2015b (www.mathworks.com).

As a whole, a very peculiar pattern is noted in RH2+ for stratiform and convective clouds, wherein, 2.6% increase in spatial coverage of stratiform clouds lead to 13% increment in VEF, and 5% increment in convective clouds does not seem to enhance VEF at all (Supplementary Fig. S7). Figure 2(a–d) show the normalized percentage change in cloud coverage and VEF with respect to CTL for stratiform and convective clouds as classified by RT. Analyzing the time series data of cloud coverage and VEF, similar signatures are found for RH2+, wherein, the intensification phase of the depression shows a surge in convective cloud coverage with little heating associated with it. The composite difference plots of hydrometeor mixing ratios (Fig. 3) show that throughout the simulation duration there is an enhancement (reduction) of frozen hydrometeors (above 600 hPa) for RH2+ (RH2−). This suggests that frozen hydrometeors predominantly act as a source (sink) modulator that defines the warming (cooling) signature of MDs (Supplementary Fig. 10).

In order to decipher the discrepancy in the heating patterns associated with convective and stratiform clouds, a time series analysis of vertical velocity (Pa/s), hydrometeor mixing ratio (kg/kg), cloud areal coverage (km^2)^, and VEF reveal the rationale behind the phenomenon. As the storm intensifies (1200 UTC 23 June–0000 UTC 24 June), the convective cloud coverage increases with little heat associated with it for RH2+. At those instances, weaker updrafts prevailed (Fig. 2f), facilitating downward movement of hydrometeors and melting them on the way (Supplementary Fig. S4e–h). This melting can be seen in the form of increased cloud water content between 800 to 500 hPa, thus cooling the ambient atmosphere and reducing the heat associated with convective clouds. On the other hand, stratiform clouds in RH2+ are mostly associated with stronger updrafts (mid-level), as noted during the period 1200 UTC 22 June to 0000 UTC 23 June. Increase in frozen hydrometeors lead to enhanced latent heat release, facilitating a rise in VEF, and stronger updrafts in a moist environment results in an increase in cloud water mixing ratio. RH2− is mostly dominated by weak updrafts (Fig. 2e), and any hike in convective cloud coverage is associated with stronger updrafts. A region of such updraft is seen at 0000 UTC 24 June, when an increase in convective cloud coverage is marked by enhanced snow, and graupel mixing ratio with diminished cloud water content, thereby boosting latent heat release, and enhancing VEF (Supplementary Fig. S4b,c).

Level wise correlations are computed between cloud coverage and VEF to ascertain which part of the troposphere responds the most to RH perturbations for a domain bounded by 66°E–77°E longitude and 18°N–27°N latitude. Lower tropospheric (1000–750 hPa) RH perturbations show higher sensitivity towards stratiform clouds with correlation coefficients of 0.62 (RH2−) and 0.80 (RH2+). Convective clouds are not much affected both in the lower (RH2− = 0.71 and RH2+ = 0.74), as well as in the mid-troposphere (700-500 hPa) where correlation coefficients are found to be 0.69 (0.67) for RH2− (RH2+). Very weak correlation of 0.32 (0.25) is found in the mid-troposphere for stratiform clouds in RH2− (RH2+). All these correlations are statistically significant at 95% confidence level. These results can be justified by our prior understanding that in mesoscale convective systems the convective clouds are typically embedded within or adjacent to the stratiform clouds and the ice particles that grow in the convective cells are carried to the upper levels through strong convective updrafts and then transferred to the neighboring stratiform region which then acts as the source of ice nucleation for stratiform clouds^42^. As the formation of convective cells is substantially reduced in RH2−, a notable decrease in stratiform clouds are observed.

### Surface–Precipitation Feedback

Another critical aspect of this work is to examine the surface–precipitation feedback changes brought in by RH perturbations and this is investigated for case 4 only. Assuming that the water vapor inside the domain is well mixed and vertical fluxes of precipitation and evaporation do not change much, the precipitation difference between experiments and CTL (${\rm{\Delta }}P$) can be formulated as^43–45^:

3 $${\rm{\Delta }}P=P^{\prime} -P={\rm{\Delta }}\chi (ET+IN)\,+\,\chi {\rm{\Delta }}ET\,+\,\chi {\rm{\Delta }}IN\,+\,{\rm{\Delta }}\chi ({\rm{\Delta }}ET+{\rm{\Delta }}IN),$$

where $\chi$ is precipitation efficiency given by

4 $$\chi =\,P/(ET+IN).\,$$

$ET$ and $IN$ represent evaporation and moisture influx respectively, with ${\rm{\Delta }}$ representing the change in experiment with respect to CTL. The first term on the right-hand side of equation (3) is the efficiency effect (*EE*) that accounts for changes in precipitation due to precipitation efficiency changes. Precipitation efficiency $\chi$ is defined as the amount of moisture that precipitates out from the moisture that enters the domain. The second term accounts for changes brought in due to change in evaporation and is termed as the surface effect (*SE*). Remote effect (*RE*), the third term in equation (3) shows the impact of altered moisture influx on precipitation, and the fourth term is a residual. $IN$ is calculated using Gauss divergence theorem due to the advantage that flux can be calculated across any arbitrary boundary.

**Figure 4:**
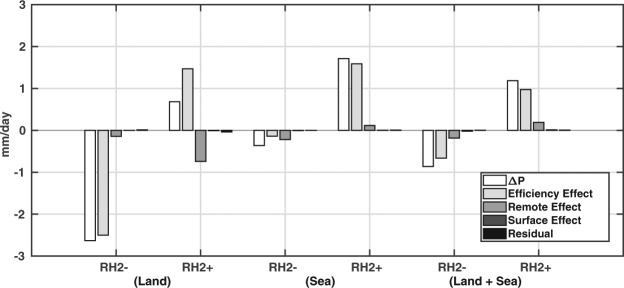
Factors affecting precipitation through efficiency effect, remote effect, surface effect, and residual, all of which sum up to ${\rm{\Delta }}P$. The analysis in which the ocean is masked is shown as Land, and Sea when land is masked. The analysis for the entire analysis domain is shown as Land + Sea. The figure is prepared with MATLAB 2015b (www.mathworks.com).

This analysis is carried out along the depression track with a 2.5° degree swath on both sides of the track, forming a capsule enclosing the storm (Supplementary Fig. S2b). Separating land and sea in the domain marked by the enclosed area, it is possible to check the impacts of perturbed RH on *P* through various pathways, both in the oceanic and the continental regimes. Overall results show *EE* as the dominating factor for changing *P* (Fig. 4). Since *EE* is controlled by precipitation efficiency change, the effect incorporates the contributions of both *IN* and *ET*. Over land, a parched lower troposphere coupled with reduced evaporation rates and depleting soil moisture (Supplementary Fig. S8) created a hostile environment for convection in RH2−. Even though RH2− has greater *IN* (149.18 mm/day) than RH2+ (146.05 mm/day), a lesser $\chi$ (0.17) lead to an overall decline in precipitation over land. On the contrary, enhanced evaporation rates in RH2+ coupled with increased soil moisture resulted in a better $\chi$ (0.20), thereby increasing precipitation over land. RE shows negative feedback on *P* in RH2+ due to a reduction in *IN* as compared to CTL (149.93 mm/day). The feedbacks act in a similar fashion over the AS, except *RE*. Suppressed *IN* (189.47 mm/day) in RH2− leads to a negative *RE* feedback, whereas, enhanced *IN* (191.63 mm/day) in RH2+ contributes to the total precipitation over the sea. Analyzing the capsule enclosing the storm track, a clear signature is noted, wherein, all the effects suppress *P* in RH2−, and enhance *P* in RH2+. This can be viewed as the cumulative effect of the feedback pathways over sea and land. *RE* shows a positive feedback even though it is negative over land in RH2+ due to a relatively greater contribution of *IN* over the sea (191.63 mm/day) than land (146.05 mm/day), thus contributing to total precipitation as a whole (Fig. 4).

## Conclusions

As the projections reckon a steady RH in the near future globally, we found a steady increase over the Indian subcontinent in the past 39 years (Supplementary Fig. 2a). Following this increasing trend, it is essential to understand how MDs would respond across a range of moisture availability. A series of surrogate simulations (4 MDs) are conducted for depressions over the BoB and the AS, wherein RH is altered in the troposphere till 500 hPa by ±2% (RH2±), and compensated for the change in the levels above, so that the vertically integrated water content remains conserved as the reference simulation (CTL). The composite analysis suggests that there is a persistent warming (cooling) of ±0.25 °C throughout the troposphere for RH2± with respect to CTL. These variations in temperatures are attributed to enhanced (suppressed) heating from the clouds.

In-depth analyses of case 4 suggested that the lack of moisture in the low–mid troposphere in RH2− suppressed the formation of both convective and stratiform clouds, which in turn lowered the VEF associated with it. On the other hand, RH2+ showed 2.6% increase in stratiform clouds, contributing 13% more VEF, but 5% increase in convective clouds did not have any impact on VEF. This disparity is associated with vertical velocity alterations throughout the simulation period. Whenever there is an increase in the convective cloud coverage, it is associated with weaker updrafts, subsequently bringing down the frozen hydrometeors. This aggravated the melting process and in turn cools the troposphere, thus reducing the heat associated with convective clouds. An antonym analogy justifies the heat released by stratiform clouds during the simulation. Further, stratiform heating showed greater sensitivity towards RH perturbations in the lower troposphere (1000–750 hPa), though convective heating showed very less sensitivity both in the low (1000–750 hPa), as well as the mid-troposphere (700–500 hPa).

Again, analyzing the surface–precipitation feedback for case 4, it is found that RH2− facilitates more *IN* than RH2+ over land, but do not contribute to precipitation due to a weaker $\chi$. On the contrary, enhanced evaporation rates in RH2+ along with increased soil moisture resulted in a better $\chi$ and enhanced precipitation over land. Overall, it is found that *EE* and *RE* are the dominating pathways through which precipitation is modulated with negligible contribution from *SE*. Even though VEF and surface–precipitation feedback analysis is carried out for case 4 only, a general consensus amongst the composite plots gave us confidence in our findings. In addition, computationally expensive calculation of VEF restrained us from analyzing further cases [Supplementary text]. However, a steady increase of RH over the Indian subcontinent in the last 39 years is indicative of a changing climate regime, and the surrogate climate simulations become a necessity to investigate the impact of these changes.

Overall we conclude that under a moist low–mid troposphere over the Indian subcontinent, stratiform clouds are the dominating means through which heat is released into the troposphere that can facilitate intense depressions with enhanced precipitation. In a nutshell, it is inferred that under moist (dry) tropospheric situations, it is highly likely that intense (weak) MDs will occur in the near future over the region.

## Method

### Model and Experiment Design

In order to evaluate the sensitivity of monsoon depressions to atmospheric humidity, a regional cloud resolving model is used [Supplementary Text]. Weather Research and Forecasting model version 3.8.1^46^ is implemented in a nested configuration for four MD cases as provided in Table 1. The simulation domains along with India Meteorological Department (IMD) storm tracks are shown in Supplementary Fig. 1.

**Table 1:** Information about MDs simulation period

Case No.	Simulation Period		Basin Name
	Start	End	
1.	0000 UTC 25-Jul-2003	0000 UTC 28-Jul-2003	BoB
2.	0000 UTC 02-Aug-2006	0000 UTC 05-Aug-2006	BoB
3.	0000 UTC 15-Sep-2008	0000 UTC 18-Sep-2008	BoB
4.	0000 UTC 22-Jun-2015	0000 UTC 25-Jun-2015	AS

All the results illustrated are for the innermost domain having a resolution of 3Km. Twelve simulations are carried out for four MDs and each set of experiment comprises of (1) a reference run (CTL), (2) a perturbed run with 2% decrement in RH till 500 hPa (RH2−), compensated by an increment in the upper levels so that the total integrated value remains constant, and (3) similar to (2) but with 2% increment till 500 hPa (RH2+). All the perturbations are governed by equation (5) where *R*_1_ refers to the unperturbed RH profile, and *R*_2_ refers to the perturbed profile. A composite of the initial vertical profile of RH and frequency distribution of climatological RH over the Indian region are present in Supplementary Fig. 12(a,b).

5 $$d\,=\,{\int }_{1000}^{50}{R}_{1}(p).dp-{\int }_{1000}^{50}{R}_{2}(p).dp\,\approx 0$$

6 $${R}_{2}(p)=\{\begin{array}{ll}(1\pm 0.02)\times \,{R}_{1}(p), & \,p\ge 500\,hPa\\ a\,\times \,{R}_{1}(p)\,, & \,p < 500\,hPa\end{array}$$

where,

7 $$a\,=\,\frac{{\int }_{500}^{50}{R}_{1}(p).dp\,\mp \,0.02\,\times \,{\int }_{1000}^{500}{R}_{1}(p).dp}{{\int }_{500}^{50}{R}_{2}(p).dp}$$

is a scale factor which compensates for the increment/decrement done till 500 hPa and is computed iteratively such that $d\to 0$ in equation (5).

The perturbation limits were decided by analyzing the time series of RH (1979–2017) over the simulation domain which showed ~2% increase in the mid-tropospheric RH since 1979, thus giving a first guess to mimic a near future scenario. The reason behind selecting these depressions was its occurrence over a homogeneous region of enhanced climatological RH (Supplementary Fig. S2).

### Rain Type (RT) Categorization Algorithm

In this study we have used Powell’s^41^ algorithm to categorize precipitation based on radar reflectivity^47,48^ and based on these identified cloud categories over the domain, corresponding pixels are obtained and averaged for each time step to arrive at mean values (Fig. 2a–d). The hydrometeor mixing ratio as shown in Fig. 3 are storm relative domain averaged values [Supplementary Text]. RT is capable of identifying shallow convective elements which play a critical role in the tropical environment. Shallow convective clouds not only heat the lower troposphere, aiding moisture to be deposited at higher levels but also play a critical role in the transition from shallow to deep convective regimes^49,50^. RT defines a mixed class for clouds near convective cores, as they may exhibit vertical motion and latent heating characteristics of either convective or stratiform clouds, or both. RT considers a grid point to be convective if it exceeds a threshold value ${Z}_{th}$(42 dBZ) or background reflectivity ${Z}_{bg}$ by ${Z}_{cc}$ defined as:

8 $${Z}_{cc}=a\,cos(\frac{\pi {Z}_{bg}}{2b}),$$

where ${Z}_{bg}$is the mean equivalent reflectivity of all the grid points within the radius of influence ${R}_{bg}$(5 km). *a* (20) and *b* (40) are user defined parameters that depend on the spatial resolution of the dataset. The maximum search radius for convective cores is limited by ${R}_{conv}$(10 km), and will only be labeled as a convective core if the grid point is within a radius ${R}_{adj}$ defined as:

9 $${R}_{adj}=\{\begin{array}{ll}{R}_{conv}, & \,{Z}_{bg}\ge {Z}_{conv}\\ {R}_{conv}-1\,km, & \,{Z}_{conv} > {Z}_{bg}\ge {Z}_{conv}-5\,dBZ\\ {R}_{conv}-2\,km, & \,{Z}_{conv}-5\,dBZ > {Z}_{bg}\ge {Z}_{conv}-10\,dBZ\\ {R}_{conv}-3\,km, & {Z}_{conv}-10\,dBZ > {Z}_{bg}\ge {Z}_{conv}-15\,dBZ\\ {R}_{conv}-4\,km,\, & \,{Z}_{bg}\le {Z}_{conv}-15\,dBZ\end{array}$$

For small echo elements, a new minimum threshold for convective classification ${Z}_{newth}\,$is set which is a function of areal coverage of the echo object.

10 $${Z}_{newth}=\{\begin{array}{ll}{Z}_{shallow}, & {A}_{low}\le A < {A}_{med}\\ {Z}_{shallow}+(\frac{A}{{A}_{high}-{A}_{med}})({Z}_{th}-{Z}_{weak}), & \,{A}_{med}\le A\le {A}_{high}\\ {Z}_{th}, & A > {A}_{high}\end{array}$$

where *A* is the echo object area, ${A}_{low}$(6 km^2^), ${A}_{med}$(50 km^2^), ${A}_{high}$(2000 km^2^), ${Z}_{shallow}$(28 dBZ), and ${Z}_{weak}$(7 dBZ) are user specified parameters. Any echo object whose areal coverage lies between ${A}_{low}$ and ${A}_{high}$ with reflectivity exceeding ${Z}_{newth}$ or ${Z}_{th}$ are categorized as ISO_CON_CORE, and objects with reflectivity less than ${Z}_{newth}\,$are categorized as ISO_CONV_FRINGE. A detailed list of cloud classification is given in Supplementary Table S1.

## Electronic supplementary material

Supplementary Information

Response to Quality Check
