# Publisher Correction: Ramifications of Atmospheric Humidity on Monsoon Depressions over the Indian Subcontinent

**DOI:** 10.1038/s41598-018-29835-3

**Published:** 2018-08-09

**Authors:** Himadri Baisya, Sandeep Pattnaik, Vivekananda Hazra, Anshul Sisodiya, Deepika Rai

**Affiliations:** School of Earth, Ocean, and Climate Sciences, Indian Institute of Technology, Bhubaneswar, Odisha India

Correction to: *Scientific Reports* 10.1038/s41598-018-28365-2, published online 02 July 2018

This Article contains a typographical error in the Results and Discussion section in the second subheading where,

“Vertical Eddy Flux (VEF)ss”

should read:

“Vertical Eddy Flux (VEF)”

