# Supplementary material for: Ramifications of Atmospheric Humidity on Monsoon Depressions over the Indian Subcontinent
Source: Sci Rep. 2018 Jul 2;8:9927. doi: 10.1038/s41598-018-28365-2 (PMC6028638; doi:10.1038/s41598-018-28365-2)
Supplement: Supplementary file 1 — Supplementary Information [file 41598_2018_28365_MOESM1_ESM.docx]

**Supplementary Information for:**

**Ramifications of Atmospheric Humidity on Monsoon Depressions over the Indian Subcontinent**

**Himadri Baisya^1^, Sandeep Pattnaik^1*^, Vivekananda Hazra^1^ , Anshul Sisodiya^1^ and Deepika Rai^1^**

^1^School of Earth, Ocean and Climate Sciences, Indian Institute of Technology Bhubaneswar, Odisha, India

*Corresponding Author, Email: [spt@iitbbs.ac.in](mailto:spt@iitbbs.ac.in), Phone: +91 7008619392

**Supplementary text**

**Model Configuration**

Weather Research and Forecasting model version 3.8.1^1^ is used for all twelve simulations. The model is configured with two nested domains of 9 km and 3 km grid spacing. The model uses 35 vertical levels with the top at 50 hPa. Model physics include Yonsei University Planetary Boundary Layer^2^, Goddard Shortwave Radiation^3^, Noah land surface model^4^, and Rapid Radiative Transfer Model Longwave Radiation^5^, along with Kain-Fritsch convection^6^, except for the inner domain where explicit convection is used. WDM6^7^ microphysics scheme is used due the advantage that it not only predicts the mixing ratio of the hydrometeors, but also their number concentrations (distribution), enabling the mean diameter to evolve in contrast to a single moment approach. The National Centre for Environmental Prediction’s (NCEP) final analysis (FNL) is used as initial and lateral boundary conditions for carrying out the simulations. Lateral boundary conditions for the outer domain are updated at 6 hours interval. Real-time global sea surface temperature^8^ analysis data is used to force the model with realistic SST every 24 hours.

**Storm Relative Compositing Technique**

All the composites are created using a 5˚ radius window following the storm as defined in India Meteorological Department’s (IMD) best track data. IMD’s track data is chosen in the light that the spread of the simulated track do not deviate much at most instances from observations and a 5˚ window captures the storm dynamics in great detail. Once the storm center is found, the data is reoriented to point towards north and this is carried out for every time step on each pressure level. Finally, we take spatial average to get the composite output in pressure Vs time coordinate for CTL, RH2-, and RH2+ respectively.

**VEF Calculation Method**

Vertical eddy transport of total heat or VEF calculation is based on eddy covariance method. For calculating VEF the steps are as follows:

**Step 1:** Each experiment is run with specific output variables for calculating VEF at 36 sec interval (1 TB each, totaling 3 TB for 3 experiments).

**Step 2:** A MATLAB post processing unit is solely developed to bring the variables from model’s terrain following eta levels to pressure levels and to nullify terrain effect on the fly, so as to save memory.

**Step 3:** 30 minutes consisting of 50 time steps is considered as the averaging time period, and perturbations for temperature ($T'$), geopotential height ($Z'$), water vapor mixing ratio ($Q'$), and vertical velocity ($\omega'$) are calculated based on this mean.

**Step 4:** Moist static energy (MSE) perturbation is calculated as $h^{'}= C_{p}T^{'}+gZ^{'}+ L_{v}Q'$, and $h^{'}\omega^{'}$ is calculated which shows MSE perturbations with vertical velocity perturbations.

**Step 5:** Finally $h^{'}\omega^{'}$ is averaged over the averaging time window (30 min) to get $\bar{h'\omega'}$. The derivative of $\bar{h'\omega'}$ with respect to pressure gives us VEF.

**Step 6:** The output is written to a NETCDF file for further analysis (3 GB each, totaling 9GB of data for all three experiments).

**Supplementary Figures**


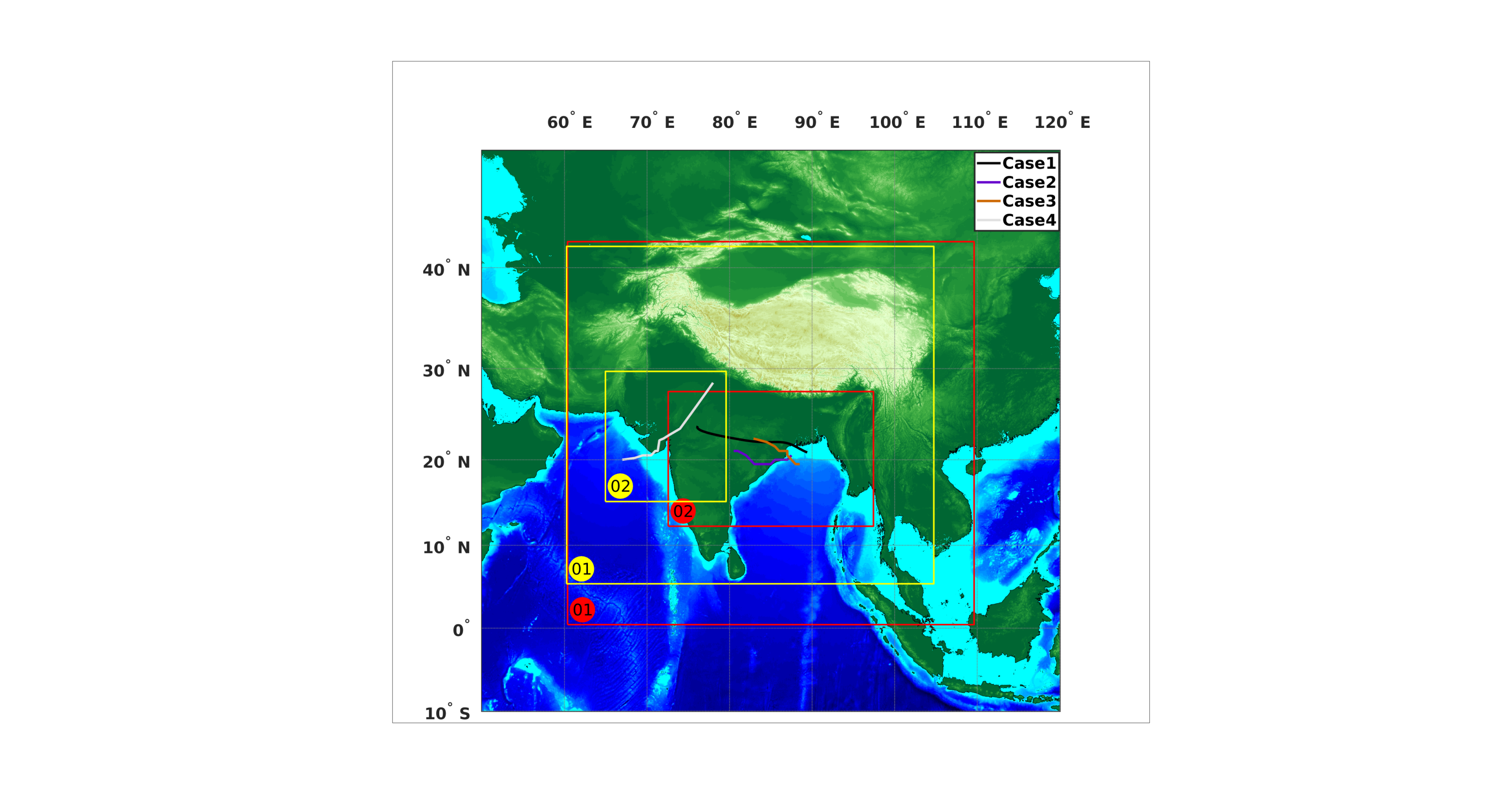


Supplementary Figure 1. Model domain configuration for four monsoon depressions along with tracks obtained from IMD’s best track data. The red (yellow) domains represent simulation of MDs over Bay of Bengal (Arabian Sea). Map is prepared with MATLAB 2015b ([www.mathworks.com](http://www.mathworks.com))

| 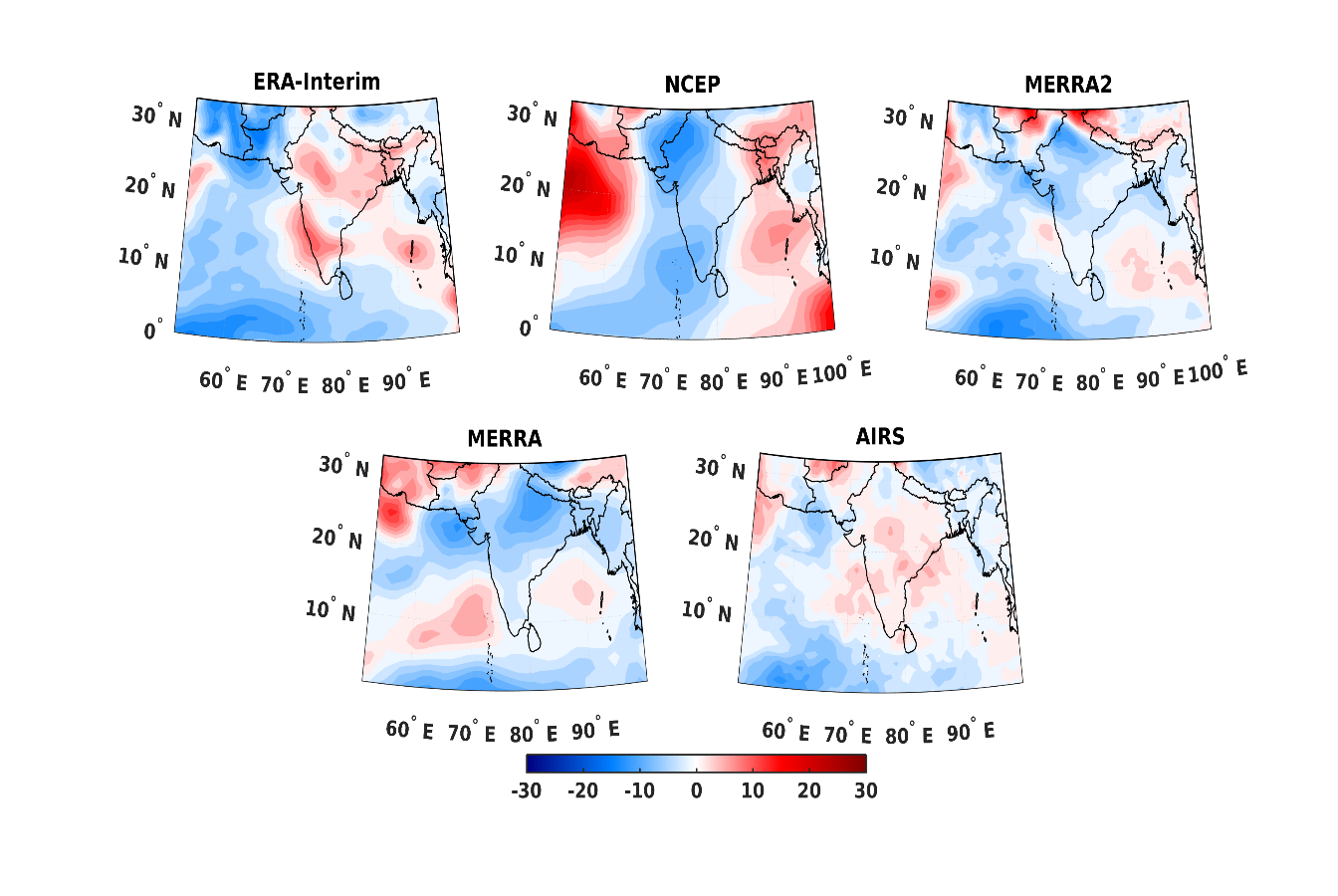  (a)  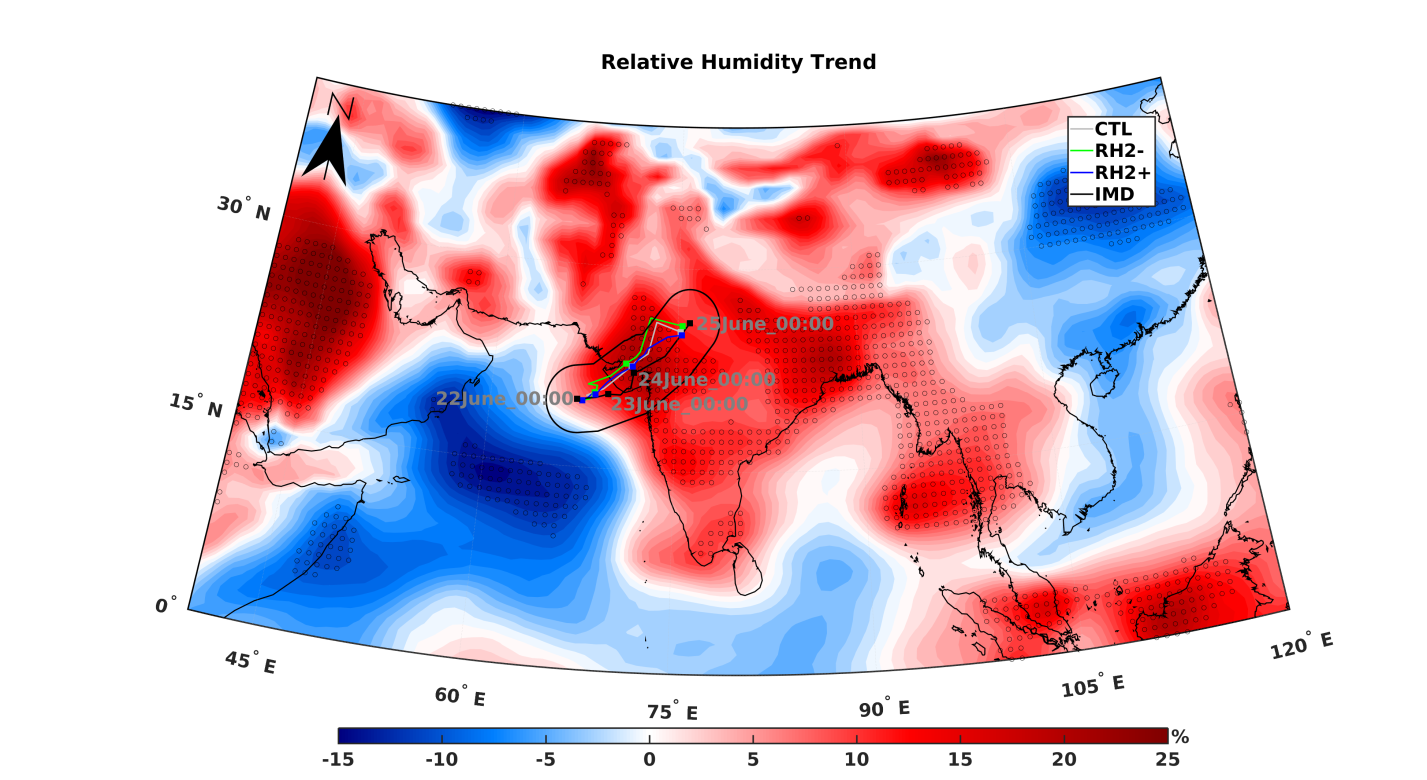  (b)  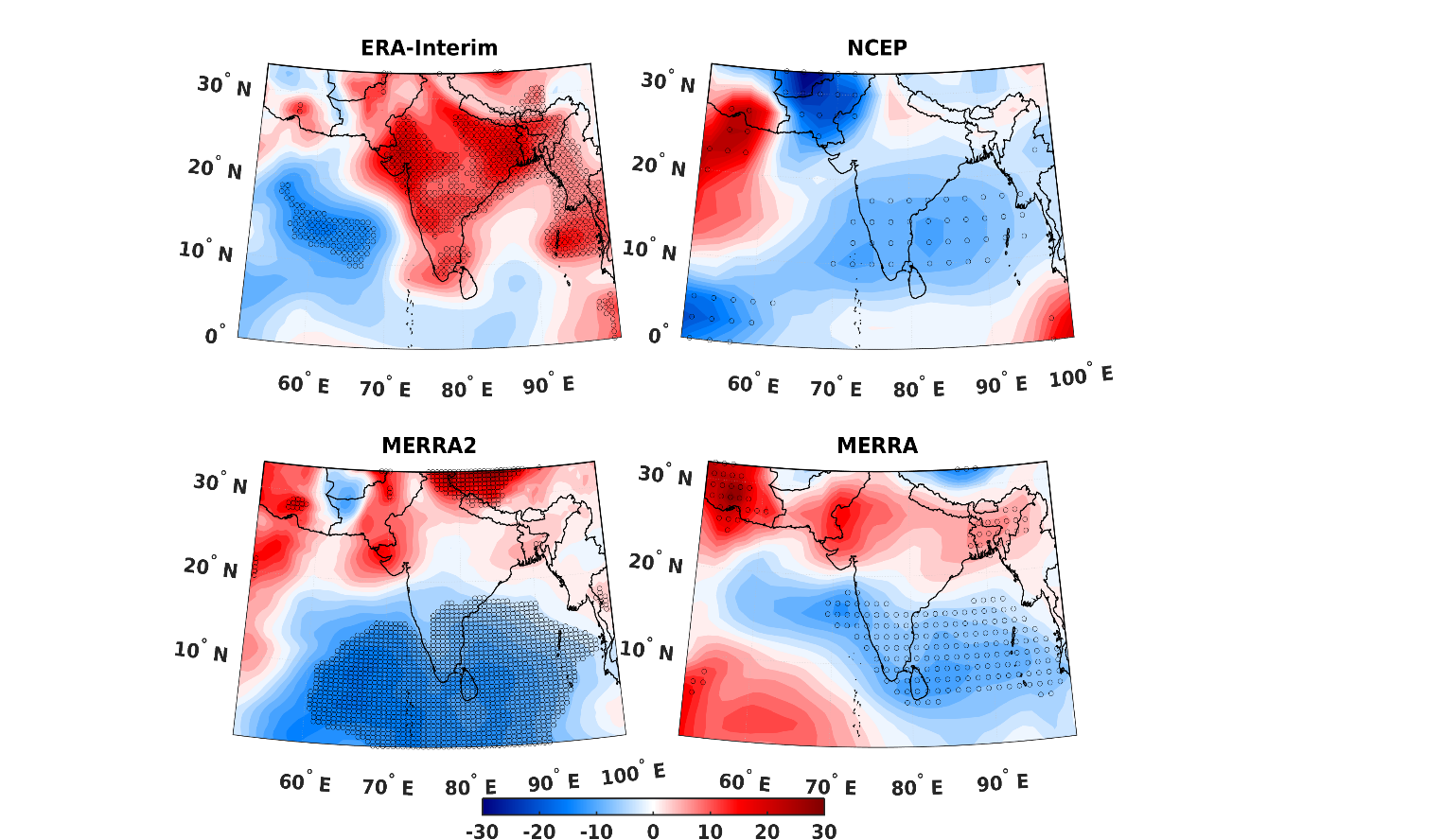  (c) |
| --- |
| **Supplementary Figure 2.** (a) Spatial trend of Mid tropospheric RH for different analysis (i.e. MERRA, MERRA-2, ERAI and NCEP) with observations from Atmospheric Infrared Sounders (AIRS) over the Indian region for the period of 2003-2017. (b) Track of experiments for case 4 (Arabian) along with IMD track. The shaded region shows the trend of RH (700 – 500 hPa) from 1979 – 2017 and the. The area enclosed by the capsule is used for surface–precipitation feedback analysis. The polygon is made with respect to IMD’s depression track and is fixed for all the simulations. Figure 2c is same figure 2a except the time period is 1979-2017 and AIRS observations are not included and dots signify statistical significance at 99% confidence level. Maps are prepared with MATLAB 2015b ([www.mathworks.com](http://www.mathworks.com)) |


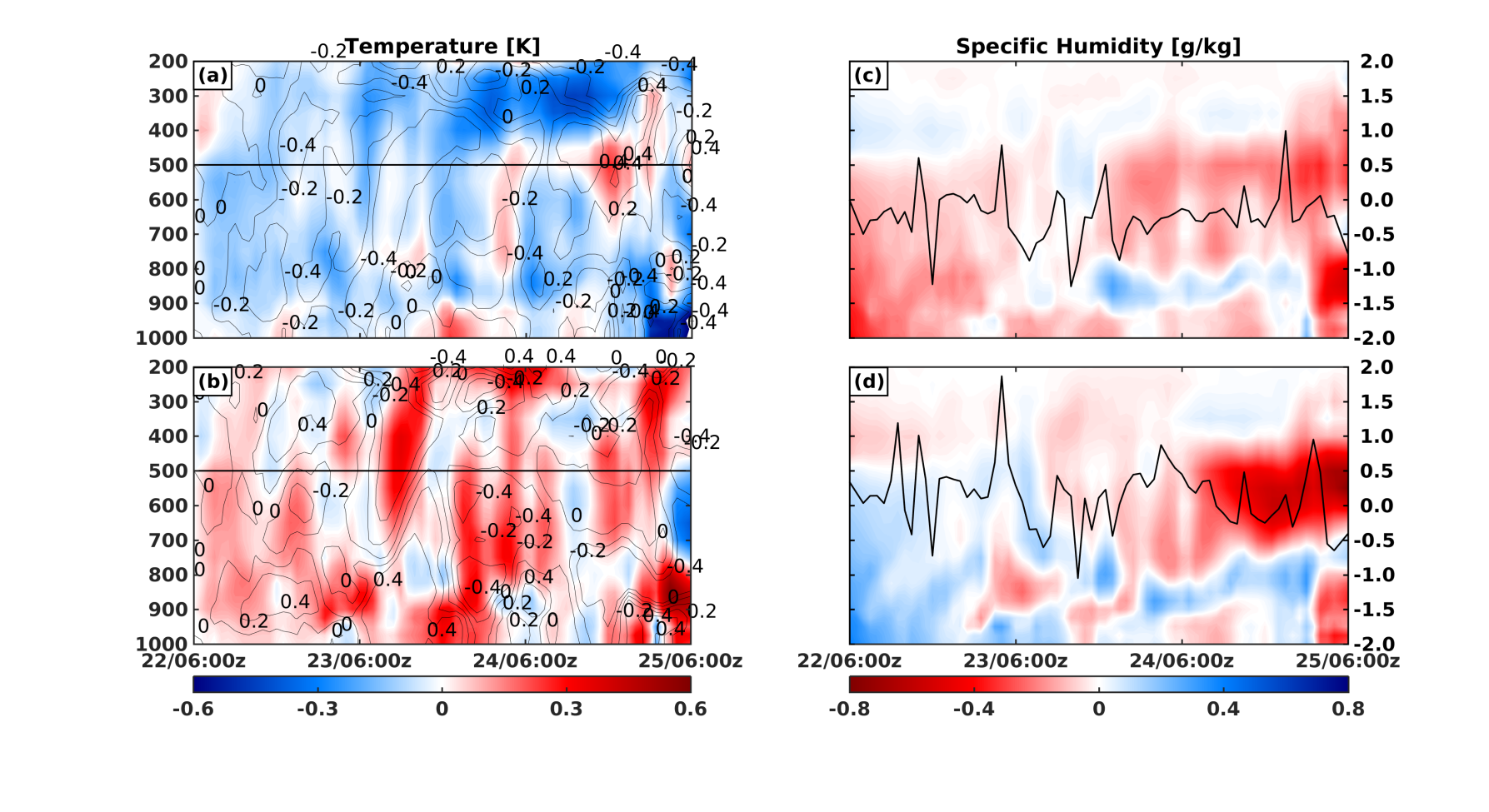


Supplementary Figure 3. Temperature (K) difference (EXP – CTL) overlaid with wind magnitude (m/s) difference contours for (a) RH2-, (b) RH2+, and specific humidity (g/kg) difference overlaid with precipitation (mm/h) difference for (c) RH2-, and (d) RH2+. The solid line at 500 hPa shows the dividing zone between perturbation and compensation of RH in the initial condition. These plots are storm relative domain averaged for case 4. Plots are prepared with MATLAB 2015b ([www.mathworks.com](http://www.mathworks.com))


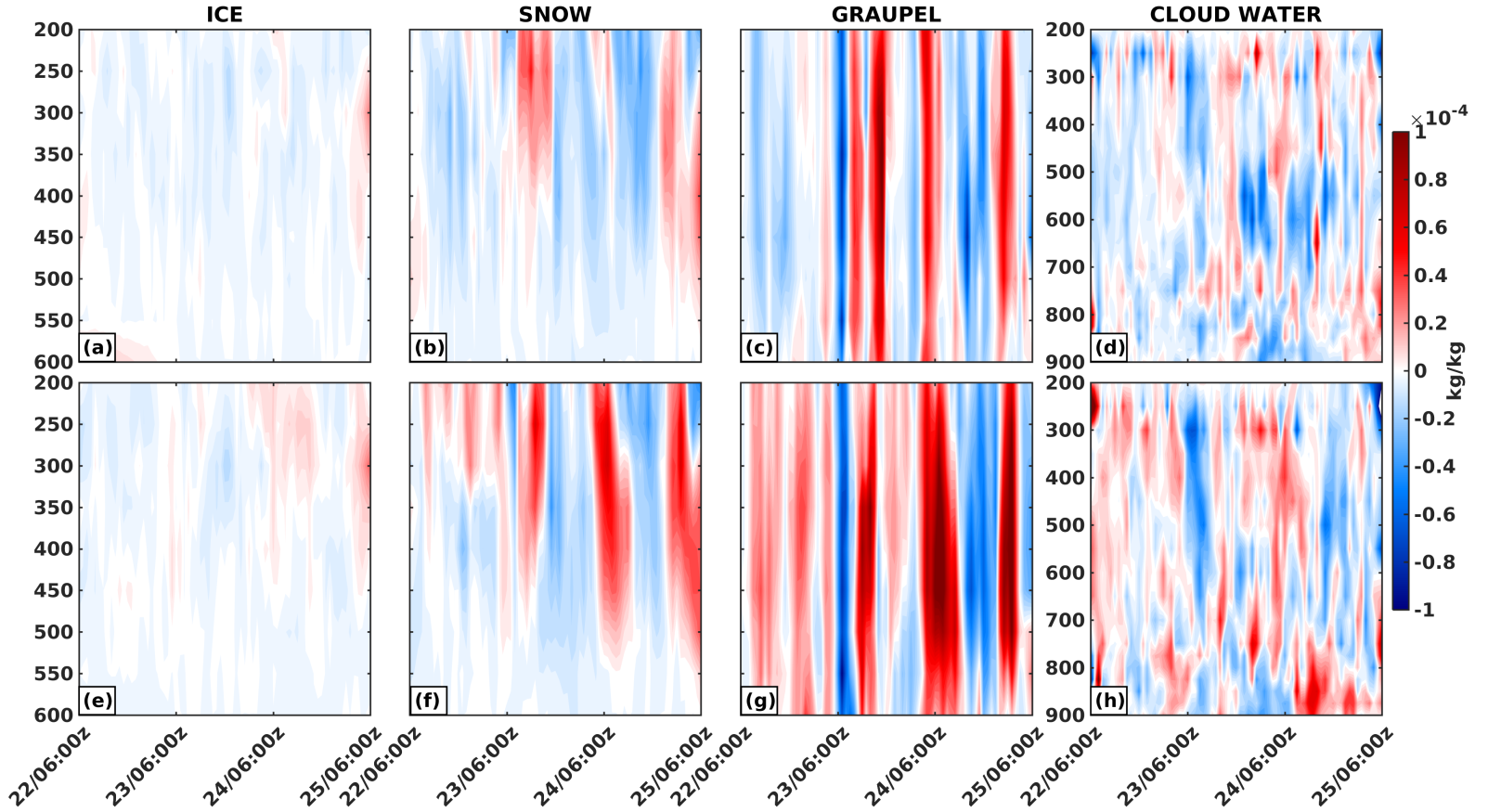


Supplementary Figure 4. Hydrometeor mixing ratio (kg/kg) difference (EXP – CTL) in RH2- for (a) ice, (b) snow, (c) graupel, and (d) cloud water. (e–h) same as (a–d) but for RH2+. These plots are storm relative domain averaged for case 4. Figures are created using MATLAB 2015b ([www.mathworks.com](http://www.mathworks.com))


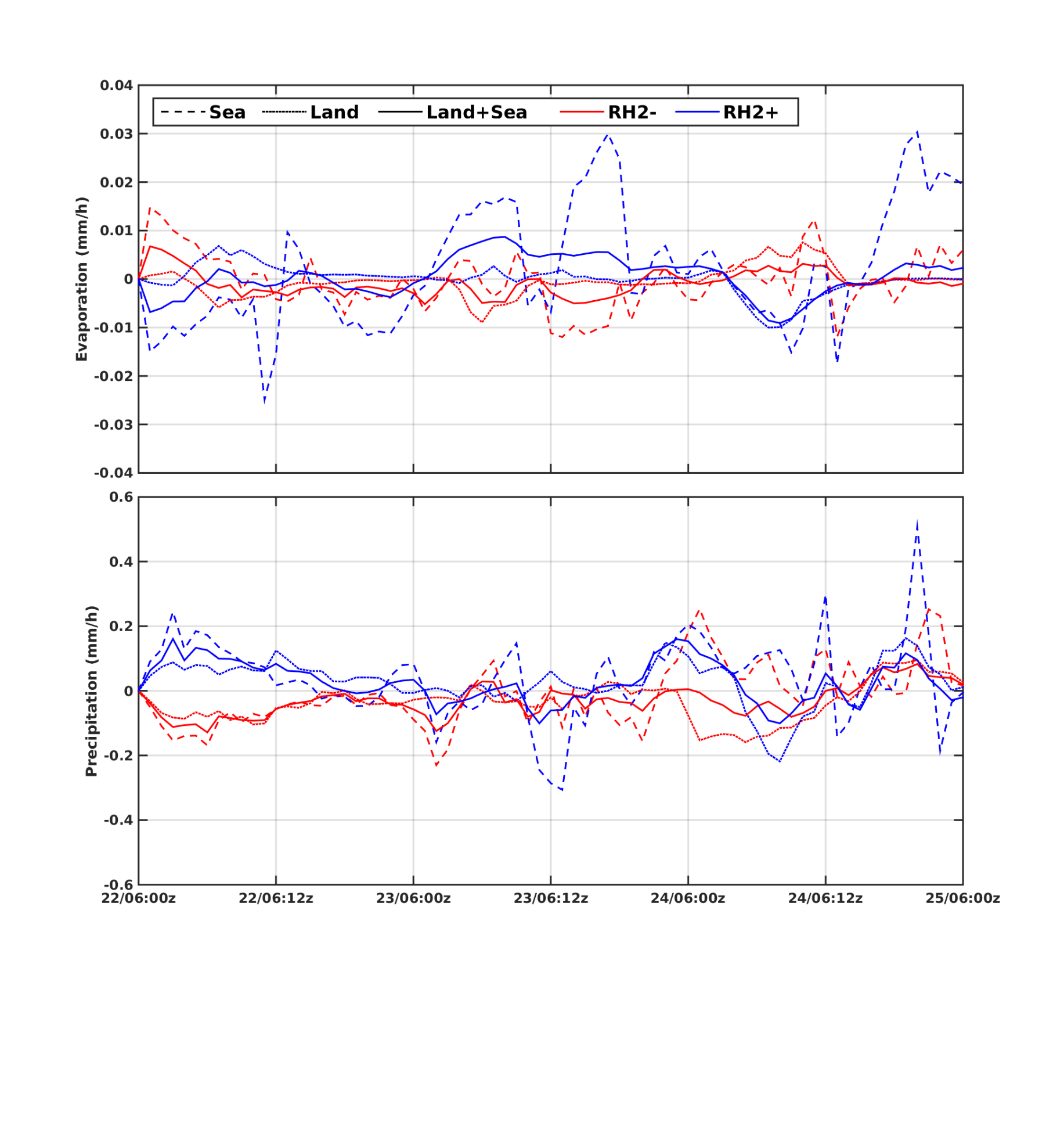


Supplementary Figure 5. Evaporation (mm/h) difference (EXP – CTL) time series for RH2- and RH2+. The dashed lines represent values over sea; dotted lines represent values over land, and the solid lines represent the entire domain average. Plot is for case 4. Figure is prepared with MATLAB 2015b ([www.mathworks.com](http://www.mathworks.com))

**(a)**


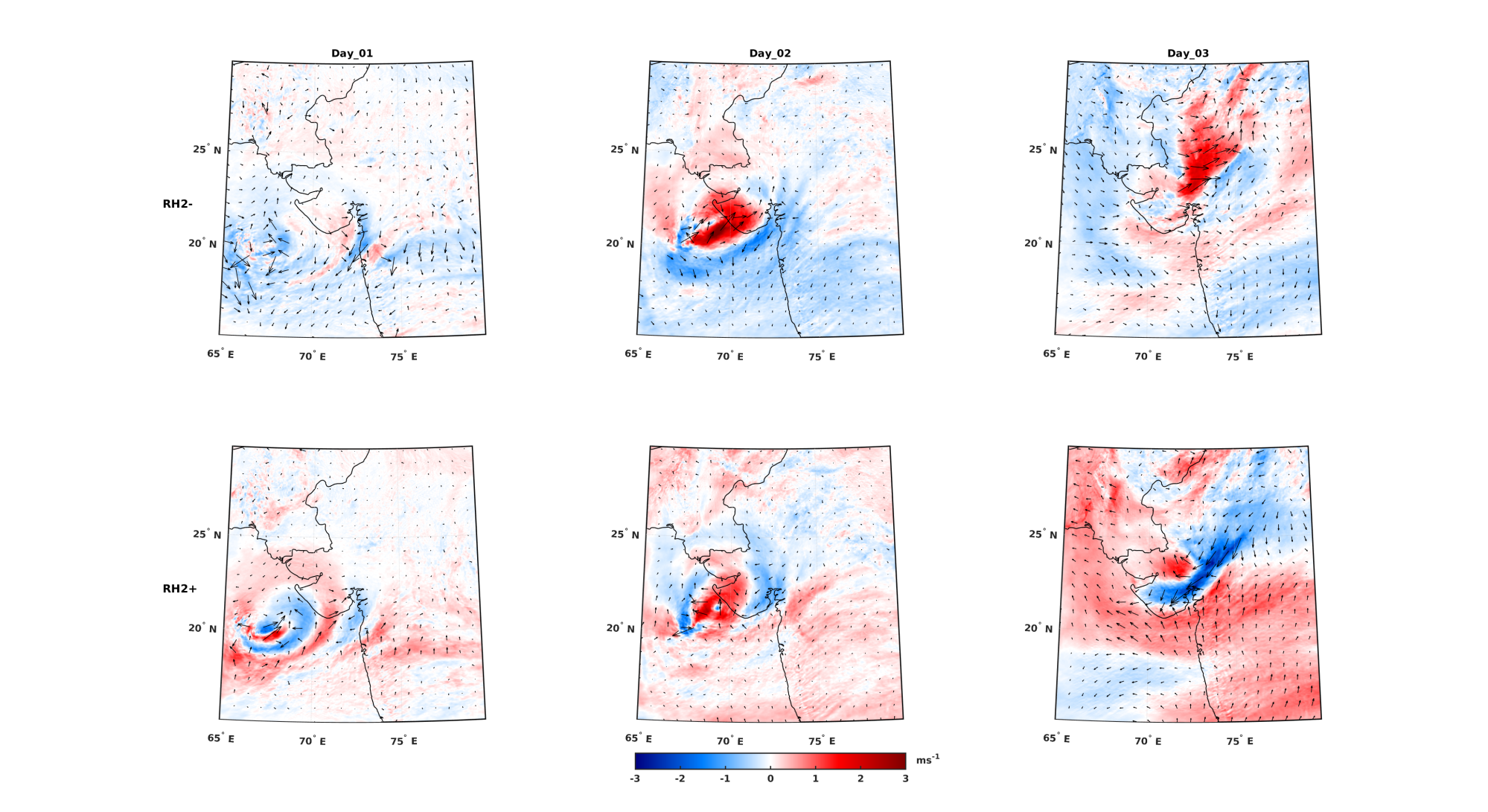


**(b)**

Supplementary Figure 6. Daily averaged 10m winds. (a) RH2-, and (b) RH2+ for case 4. Maps are prepared with MATLAB 2015b ([www.mathworks.com](http://www.mathworks.com))


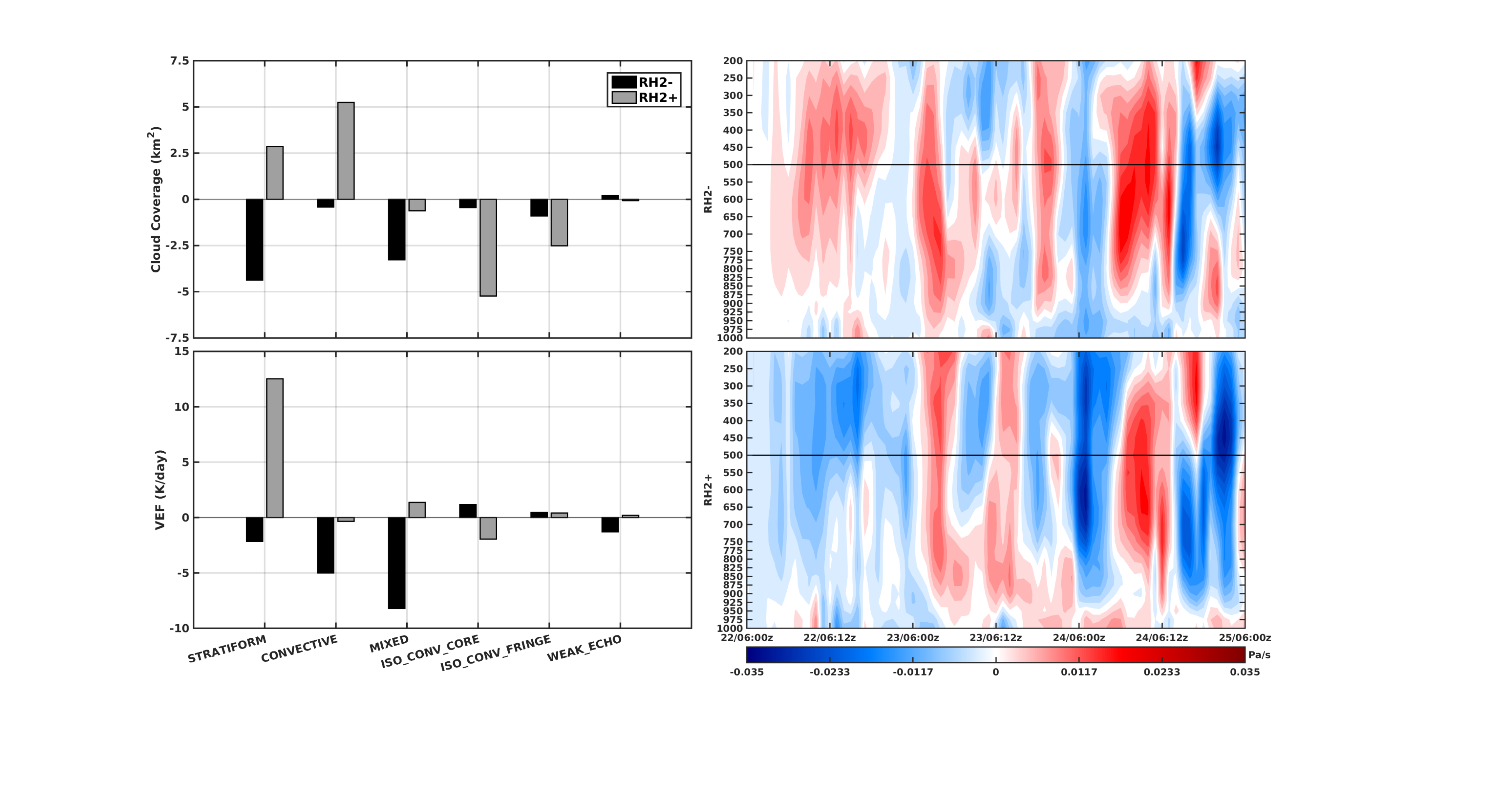


Supplementary Figure 7. Percentage change in cloud coverage (km^2^), and VEF (K/day) for all the cloud types as classified by RT. Statistics of case 4 for the entire simulation period is depicted in this plot. Figure is prepared with MATLAB 2015b ([www.mathworks.com](http://www.mathworks.com))


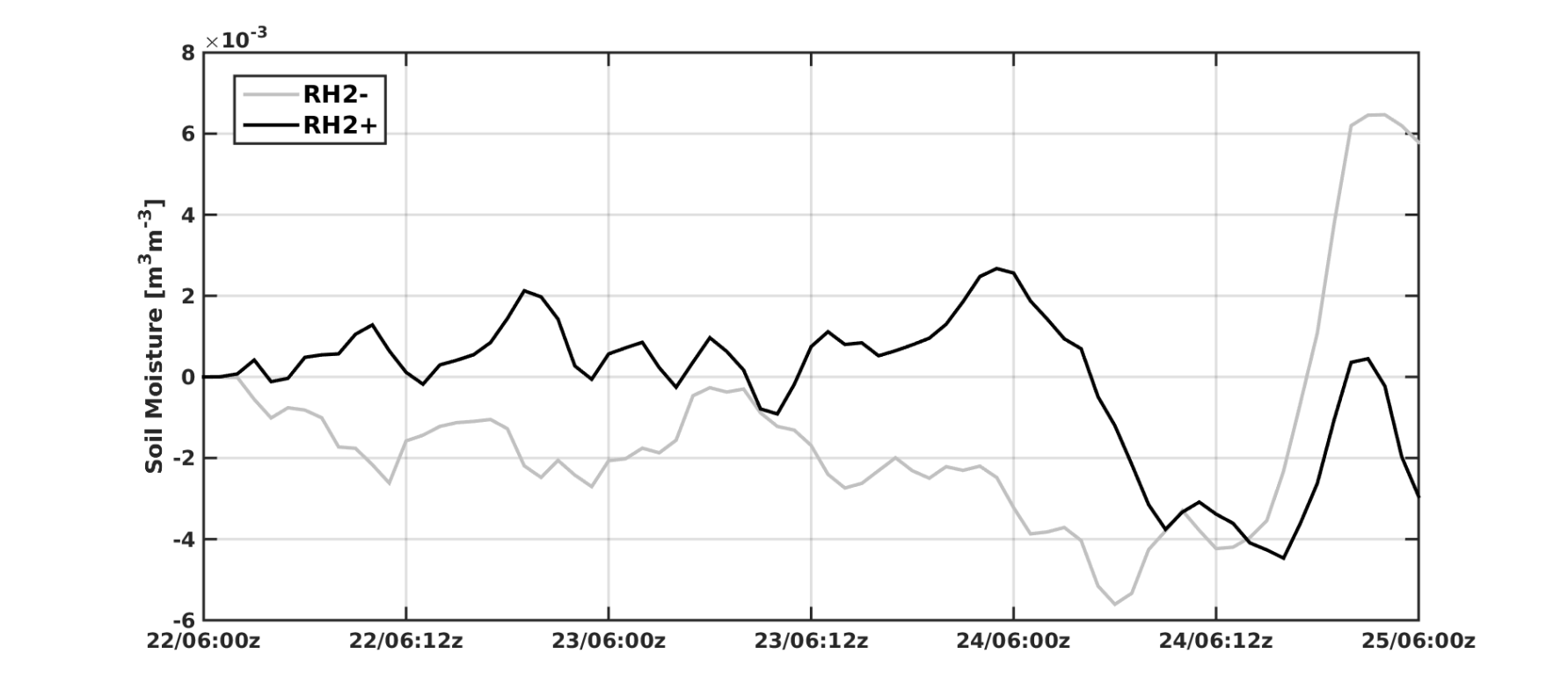


Supplementary Figure 8. Top 10 cm soil moisture difference between experiments and CTL for case 4. Figure is prepared using MATLAB 2015b ([www.mathworks.com](http://www.mathworks.com))


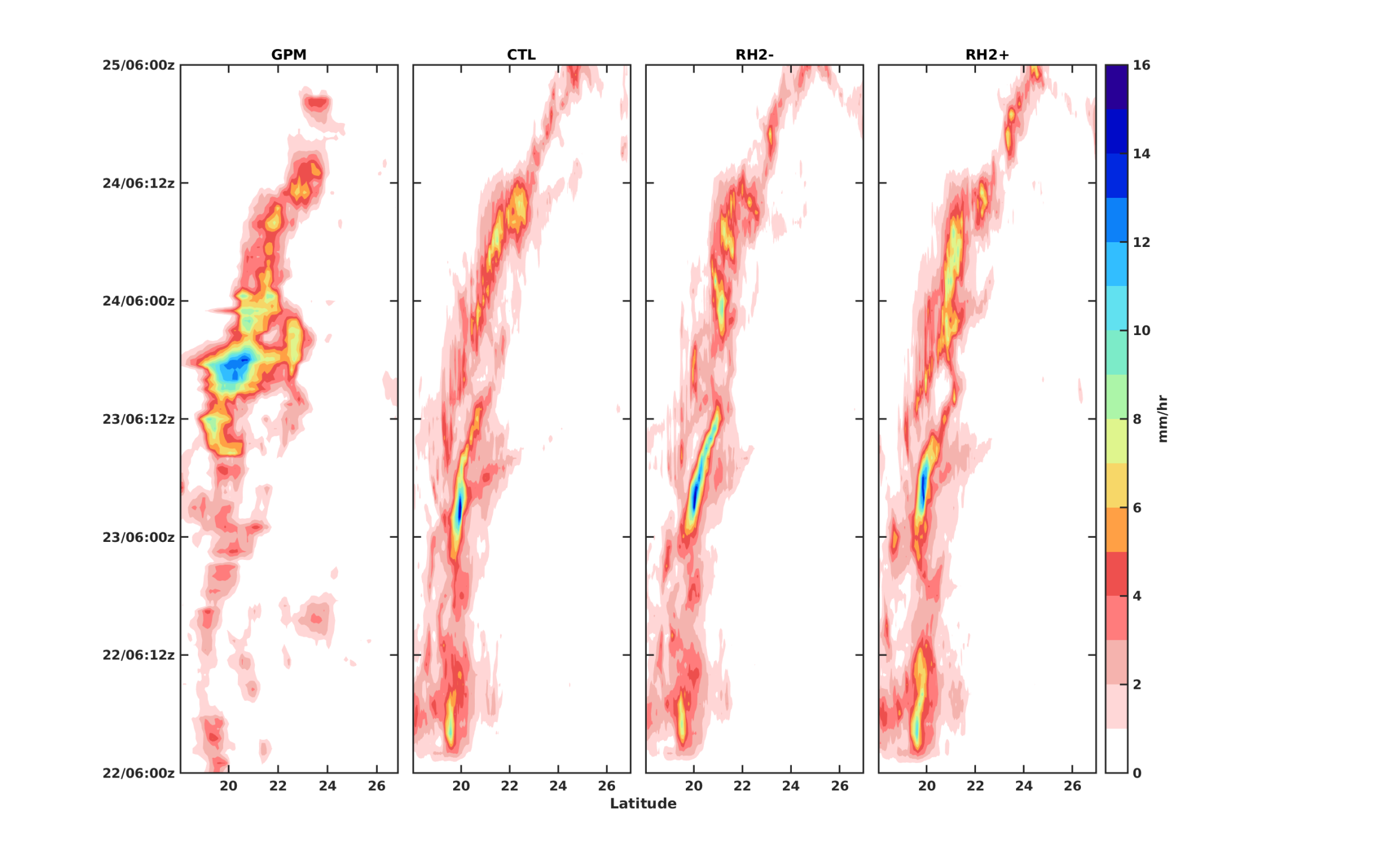


Supplementary Figure 9. Hovmoller diagram of rain rate (mm/hr) from GPM^11^, CTL, RH2- and RH2+ for case 4. Figures are created using MATLAB 2015b ([www.mathworks.com](http://www.mathworks.com))

**
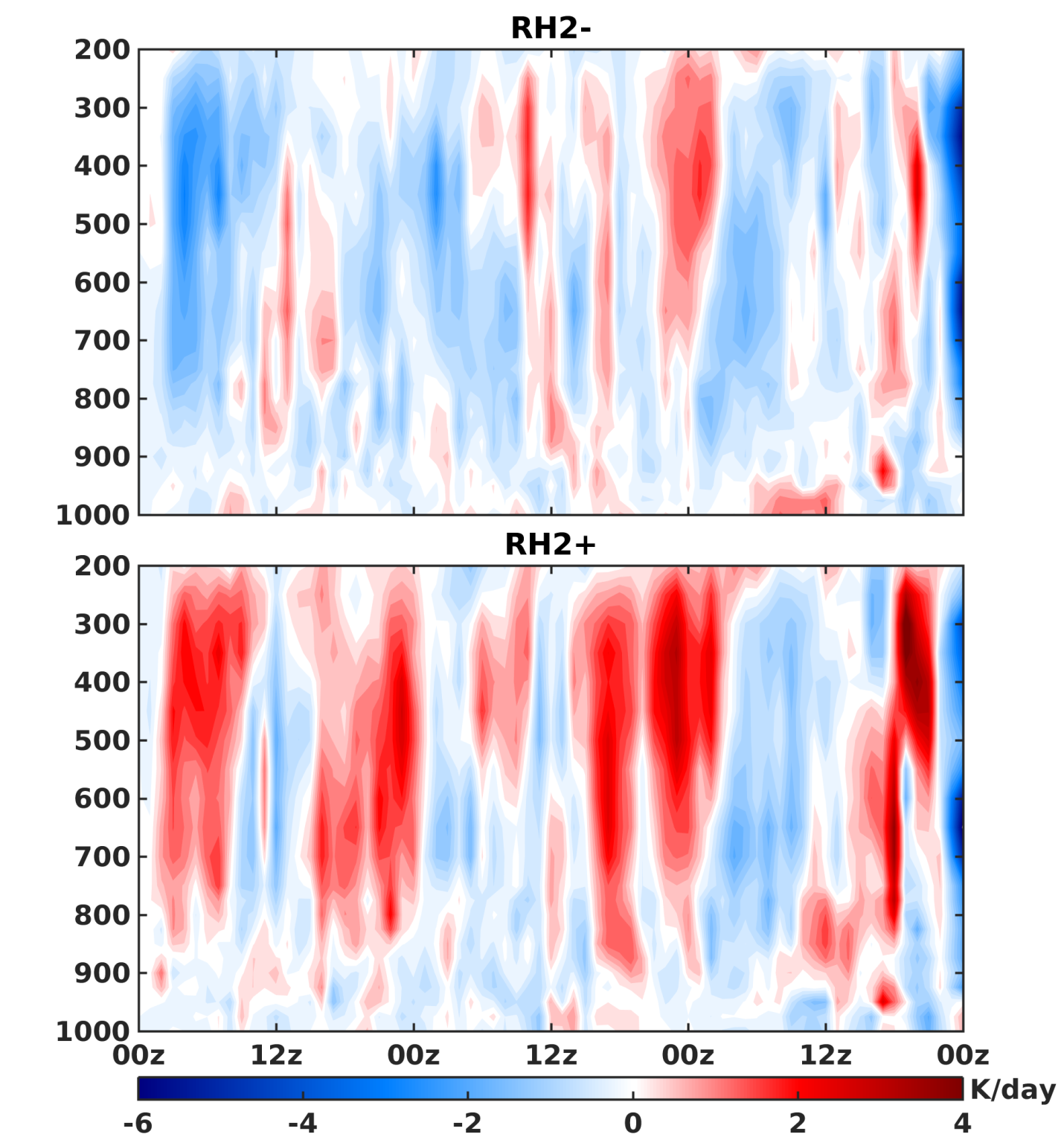
**

Supplementary Figure 10. Difference of <Q1> for case 4 (Arabian Sea) between experiments (RH2+ and RH2-) and CTL. Units are in K/Day. Figures are created using MATLAB 2015b ([www.mathworks.com](http://www.mathworks.com))


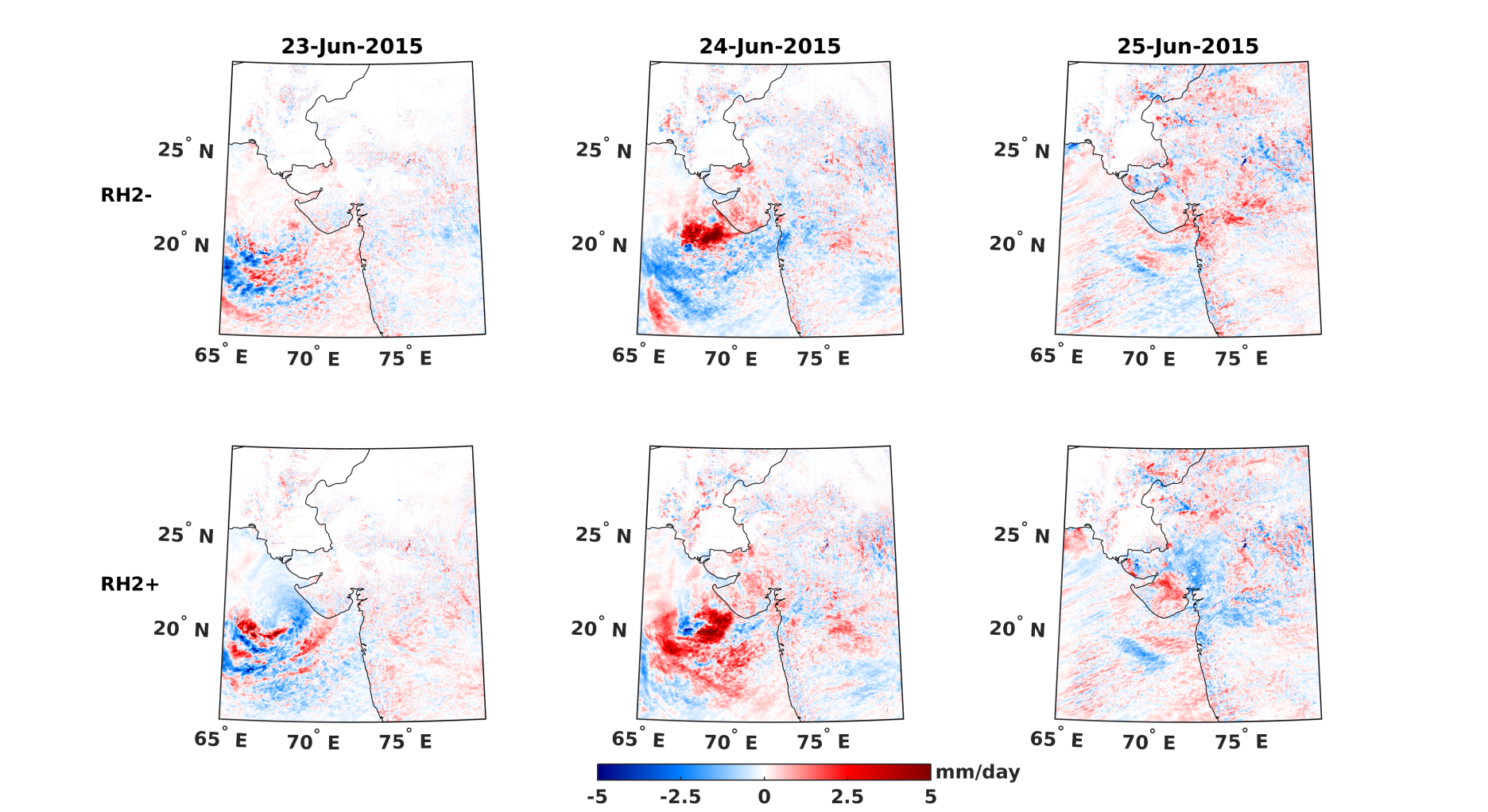


Supplementary Figure 11. Daily accumulated evaporation (mm/day) difference (EXP-CTL) for the entire simulation period for case 4 (Arabian Sea). Maps are created using MATLAB 2015b ([www.mathworks.com](http://www.mathworks.com))

| 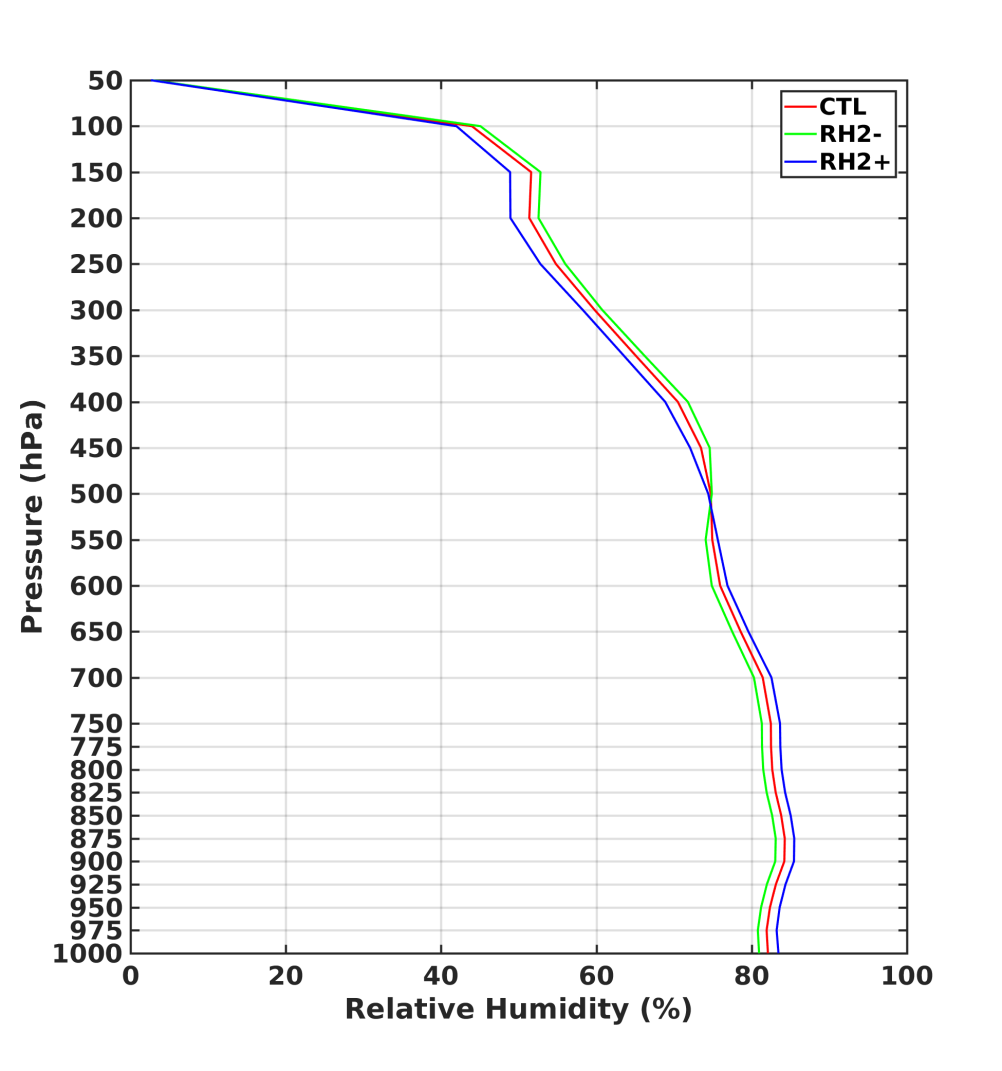 |
| --- |
| (a) |
| 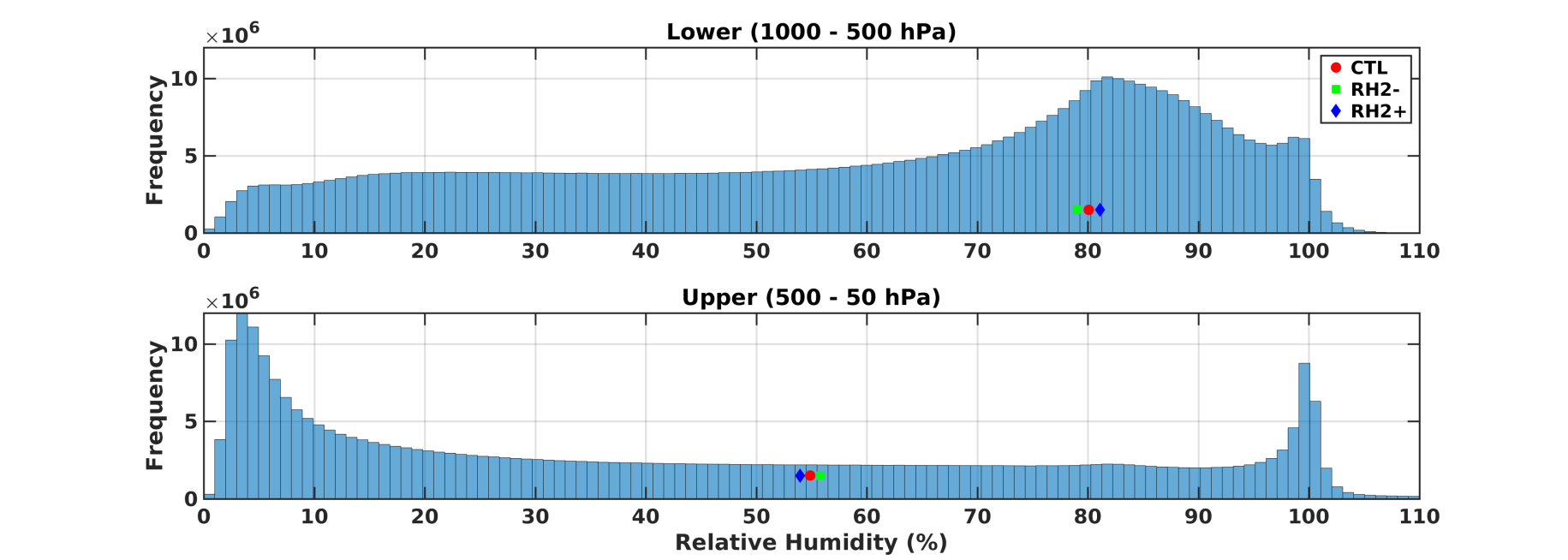 |
| (b) |
| Supplementary Figure 12. (a) Domain averaged vertical profile composite of four MDs at initial time for different experiments. (b) Frequency distribution of RH for JJAS during 1979-2017 over the Indian region (40˚E-120˚E, -5˚S-40˚N) using ECMWF^12^ interim analysis. The dots represent the composite of four monsoon depressions. Figures are created using MATLAB 2015b ([www.mathworks.com](http://www.mathworks.com)) |

**Supplementary Table S1.** List of cloud classifications made by Rain Type (RT) algorithm^13^.

| **Cloud Category** | **Description** |
| --- | --- |
| CONVECTIVE | Strongest echoes or those with reflectivity much larger than the background. Usually contain positive latent heating. |
| STRATIFORM | Weak precipitating echoes often associated with large systems. Bright band often present near 0˚C level. Latent heating maximum (minimum) in upper (lower) troposphere usually present. |
| MIXED | Surrounds convective cores. Contains echo columns that have heating characteristics of either convective or stratiform echoes, or sometimes both. Confident classification extremely difficult using only reflectivity. |
| ISO_CONV_CORE | Strongest echoes in small echo objects. Often represents the cores of developing shallow and isolated convection. |
| ISO_CONV_FRINGE | Weaker echoes in small echo objects. Includes weak, decaying convection and echoes surrounding isolated convective cores. |
| WEAK ECHO | Mostly consists of non-meteorological echo or, otherwise, very small, weak features that have little implication for latent heating. |

**Supplementary References**

S1. Skamarock, W. C. *et al.* *A Description of the Advanced Research WRF Version 3*. *Technical Report* (2008).

S2. Hong, S.-Y., Noh, Y. & Dudhia, J. A new vertical diffusion package with an explicit treatment of entrainment processes. *Mon. Weather Rev.* **134,** 2318–2341 (2006).

S3. Chou, M.-D. & Suarez, M. J. An efficient thermal infrared radiation parameterization for use in general circulation models. *NASA Tech. Memo. 104606* **3,** 102 pp. (1994).

S4. Ek, M. B. & Holtslag, A. A. M. Influence of Soil Moisture on Boundary Layer Cloud Development. *J. Hydrometeorol.* **5,** 86–99 (2004).

S5. Mlawer, E. J., Taubman, S. J., Brown, P. D., Iacono, M. J. & Clough, S. A. Radiative transfer for inhomogeneous atmospheres: RRTM, a validated correlated-k model for the longwave. *J. Geophys. Res.* **102,** 16663 (1997).

S6. Kain, J. S. The Kain–Fritsch Convective Parameterization: An Update. *J. Appl. Meteorol.* **43,** 170–181 (2004).

S7. Lim, K.-S. S. & Hong, S.-Y. Development of an Effective Double-Moment Cloud Microphysics Scheme with Prognostic Cloud Condensation Nuclei (CCN) for Weather and Climate Models. *Mon. Weather Rev.* **138,** 1587–1612 (2010).

S8. Gemmill, W., Katz, B., Li, X., Lawrence, M. & Burroughs, L. D. *The daily Real-Time, Global Sea Surface Temperature – High Resolution Analysis: RTG_SST_HR*. (2006).

S9. Martens, B. *et al.* GLEAM v3: satellite-based land evaporation and root-zone soil moisture. *Geosci. Model Dev.* **10,** 1903–1925 (2017).

S10. Miralles, D. G. *et al.* Global land-surface evaporation estimated from satellite-based observations. *Hydrol. Earth Syst. Sci.* **15,** 453–469 (2011).

S11. Acker, J. G. & Leptoukh, G. Online Analysis Enhances Use of NASA Earth Science Data. *Eos, Trans. Am. Geophys. Union* **88,** 14 (2007).

S12. Dee, D. P. *et al.* The ERA-Interim reanalysis: configuration and performance of the data assimilation system. *Q. J. R. Meteorol. Soc.* **137,** 553–597 (2011).

S13. Powell, S. W., Houze, R. A. & Brodzik, S. R. Rainfall-type categorization of radar echoes using polar coordinate reflectivity data. *J. Atmos. Ocean. Technol.* **33,** 523–538 (2016).
