# Supplementary material for: Ramifications of Atmospheric Humidity on Monsoon Depressions over the Indian Subcontinent
Source: Sci Rep. 2018 Jul 2;8:9927. doi: 10.1038/s41598-018-28365-2 (PMC6028638; doi:10.1038/s41598-018-28365-2)
Supplement: Supplementary file 2 — Response to Quality Check [file 41598_2018_28365_MOESM2_ESM.docx]

**Response to Quality Checks**

-- Please number all equations sequentially.

**Response:**

**All equations are numbered sequentially in the revised manuscript uploaded.**-- Please ensure that the main figures (excluding supplementary figures) are uploaded as separate figure files (i.e. one image file for Fig 1, one file for Fig 2...) in one of the following file formats: .jpg, .eps, .tiff or .pdf. Please note that we do not allow labels (e.g. "Figure 1") to be included in the uploaded figure image files. However, labels for parts of the figure such as “(a)” or “(b)” are fine. Additionally, please ensure that each of your separate figure files consists of a single page, rather than multiple pages.

**Response:**

**Separate image files (total 4) in .eps format are uploaded.**
-- Thank you for including a statement regarding competing ‘financial’ interests, however we require that this declaration refers to both financial and non-financial interests. Can you please therefore update this statement to refer to ‘competing interests’ and, if applicable, to also list any non-financial competing interests as outlined in our editorial policies? Please note that the Competing Interests statement on the system must match the Competing Interests statement provided in the article file. Please see the following guidelines for instructions on preparing this statement: [www.nature.com/srep/policies/index.html#competing](http://www.nature.com/srep/policies/index.html#competing)

**Response:**

**As per suggestion, following statement has been updated**

**Competing financial interest**: Authors declare there is no competing financial and non- financial interest

-- We notice that figure 4 has not been referenced in the main text of the article. If figures are not cited in the manuscript they will not appear in the html (online) version if your paper is accepted for publication. Therefore, it is essential they are mentioned at least once in the text and, we strongly recommend, in the order in which they are numbered.

**Response:**

**Fig.4 has been incorporated in the revised article.**
